# Animal Grafting—Its Therapeutical Application to Certain Lesions of the Dental Apparatus

**Published:** 1879-05

**Authors:** E. Magitot


### ARTICLE VI.

### *Animal Grafting—Its Therapeutical Application to Certain Lesions of the Dental Apparatus.*

BY DR. E. MAGITOT.

Read before the French Academy of Science, January 6, 1879, and translated for the *Missouri Dental Journal*, by F. Frizelle.

In a previous communication I presented to the Academy, in common with the regretted physiologist, Ch. Legros, cases of the grafting of dental follicles in certain species of mammals. To-day I will touch upon another problem—that of the grafting of adult dental organs, and this time the results favor experiments of a more practical nature.

Grafting, as practiced with the dental organs, is divided into several varieties. The first category comprehends the grafting of teeth taken from their own alveoli, and replaced either immediately or after an indefinite period of time. This is *grafting by restitution.* In a second group are placed those teeth which are taken from one alveolus and transplanted into another, either in the same subject or in a different one. This is *grafting by transposition* Finally, in a third category are placed cases of grafting teeth in different points of the body otherwise than the jaws. The experiments of Hunter, A. Cooper, Philipeaux, and others, are examples. This is *heteroptic·grafting.*

In the present communication I will confine myself to presenting cases of grafting by restitution, but comprehending a particular variety ; that of raising an organ from its normal connections, removing by resection the diseased part, and restoring the remaining healthy portion to its primitive place. This is a combination of grafting and resection. [The first attempt of this kind was made by Dr. Delabarre, who having removed a tooth on account of abscess and fistula, excised a part of the root and replanted it with success.—Annals du Cercle Medical, 1820, 1re partie, p. 323.—The second was that of Prof. Alquie, of Montpellier, who in 1858 cured by the same operation a fistula in the chin, of long standing.—Bulletin de Therapeutique, 30 Mars, 1858.] My experiments run back only to 1875. The first three were published at that time (Gazette des Hopiteaux 1875, p. 35 et suiv.) Others figure in the inaugural Thesis of two of my pupils, Dr. Pietkiewicz (Theses de Paris, 1876) and Dr. David (Theses de Paris, 1877.) To-day the number of these operations attains the figure of 62.

The surgical indication of grafting combined with resection rests essentially on the diagnosis of a special lesion at the apical extremity of the teeth, characterized by *chronic periostitis* at this point: that is to say, inflammation of the periosteal membrane, denudation and necrosis of the subjacent cement, and resorption of the dentine—a kind of mortification of the root. The morbid processes are manifested by the following series of accidents: Inflammation of the gum or the face, denudation, and necrosis of the alveolar border, mucous or cutaneous fistulæ, etc. These accidents sometimes take a chronic, sometimes an intermittent form, and if abandoned to themselves, may result in grave disorders, deformities and cicatrices of the face, and general affections which may endanger the life of the patient. The therapeutical remedy in case of a lesion thus defined, is the removal of the mortified apex of the root which is the inciting cause of inflammation. Now, this suppression not proving effective directly, it becomes necessary to remove the entire organ in order to effect externally the removal of the diseased portion. At this period the operation of grafting comes in, which causes the restoration of the remaining healthy portion to its primitive place.

The manipulative process comprehends three parts: 1st. Total ablation of a tooth on which chronic periostitis of the apex has been diagnosed. 2d. A surgical resection of the diseased portion. 3d. Immediate re implantation. [Incidentally between the second and third operations, the surgeon can before grafting, perform other operations: washing out the purulent collection, ablation of sequestrum, and on the tooth even, resection of certain portions of the crown, obturation in case of caries, etc.] The rest of the operation consists in the sometimes necessary application of bandages (retaining appliances,) draining of purulent collections, the removal of necrosed alveolus, etc. · But the last part of the operation is very simple. When the consolidation of the grafting is effectual, there is a slight local reaction, but little or no general phenomena; the fistulæ close, the collection dries up, and the complete consolidation is accomplished in a time varying from eight to fifteen days. When, on the contrary, the operation is unsuccessful, the grafted tooth drops out in a few days from suppuration.

The success I have had in this method of operation is shown by the following figures: 62 operations have been performed, 57 cures effected—that is to say, an average of success of about 92 per cent. [Of cures dating from two years and a half back, those of the last two years especially, figure largely in our reports. The age of the subjects does not appear to exercise any influence on the results, and all kinds of teeth have been alike subjected to this process. In a large number of cases periostitis of the apex was not accompanied by any concomitant caries; in others co existent caries was removed before re implanting the tooth.]

*Conclusions.*—1st. Chronic periostitis of the apex of the roots of teeth, complicated with neighboring lesions, inflammation, abscess, denudation, maxillary necrosis, fistulæ, simple or compound, hitherto treated by simple ablation, is not beyond the resources of conservative therapeutics.

2d. The treatment consists in the resection of the affected portion of the root after the temporary removal of the tooth, followed by its immediate re-implantation—or grafting by restitution.

3d. The result of the cure is the cessation of all disease, the firm adherence of the organ, the complete return of its vascular connections and the re-establishment of its usefulness.—*Missouri Dental Journal.*

